# Circulation of human influenza viruses and emergence of Oseltamivir-resistant A(H1N1) viruses in Cameroon, Central Africa

**DOI:** 10.1186/1471-2334-10-56

**Published:** 2010-03-08

**Authors:** Richard Njouom, Serge A Sadeuh Mba, Dominique Noah Noah, Victoria Gregory, Patrick Collins, Pierre Cappy, Alan Hay, Dominique Rousset

**Affiliations:** 1 Laboratory of Virology, Centre Pasteur of Cameroon, Yaounde, Cameroon; 2 Department of Disease Control, Ministry of Health, Yaounde, Cameroon; 3 WHO Collaborating Centre for Reference and Research on influenza, MRC National Institute for Medical Research, London, UK

## Abstract

**Background:**

While influenza surveillance has increased in most developing countries in the last few years, little influenza surveillance has been carried out in sub-Saharan Africa and no information is available in Central Africa. The objective of this study was to assess the prevalence of influenza viruses circulating in Yaounde, Cameroon and determine their antigenic and genetic characteristics.

**Methods:**

Throat and/or nasal swabs were collected from November 2007 to October 2008 from outpatients with influenza-like illness (ILI) in Yaounde, Cameroon and analyzed by two different techniques: a one-step real time reverse transcription-polymerase chain reaction (RT-PCR) and virus isolation in MDCK cells. Typing and subtyping of virus isolates was performed by hemagglutination inhibition (HI), and viruses were sent to the WHO Collaborating Centre in London, UK for further characterization and analyses of antiviral resistance by enzyme inhibition assay and nucleotide sequencing.

**Results:**

A total of 238 patients with ILI were sampled. During this period 70 (29%) samples were positive for influenza by RT-PCR, of which only 26 (11%) were positive by virus isolation. By HI assay, 20 of the 26 isolates were influenza type A (10 H3N2 and 10 H1N1) and 6 were influenza type B (2 B/Victoria/2/87 lineage and 4 B/Yagamata/16/88 lineage). Seven (70%) of the H1N1 isolates were shown to be resistant to oseltamivir due to a H275Y mutation.

**Conclusions:**

This study confirmed the circulation of influenza A(H1N1), A(H3N2) and B viruses in the human population in Central Africa and describes the emergence of oseltamivir-resistant A(H1N1) viruses in Central Africa.

## Background

Influenza is an acute, highly contagious respiratory infection that can cause very serious illness [1]. Identification and characterization of circulating influenza viruses is essential to detect the emergence of antigenic drift variants causing influenza epidemics and novel A subtypes with the potential to cause an influenza pandemic [2]. Thus influenza surveillance provides a basis for selection of the virus strains to be included in the annual formulation of influenza vaccines [3,4]. The recent emergence of new highly virulent influenza A(H5N1) viruses, their wide circulation in wild and domestic birds and the associated human infections with high mortality, has raised global concern about the risk of another influenza pandemic [5]. In response, increased monitoring of influenza and enhanced preparedness to counter an emerging pandemic are being implemented worldwide [6]. While most countries in Asia, North America and Europe have increased influenza surveillance in the last few years, little influenza surveillance has been carried out in sub-Saharan Africa [7-11] and no information is available for Central Africa. To address this deficiency, the Cameroon Ministry of Public Health implemented a national influenza surveillance system in November 2007. This report presents the results of the first year surveillance.

## Methods

### Analysis of clinical specimens

Throat and/or nasal swabs were collected by physicians from outpatients with clinical evidence of influenza-like illness (ILI), defined as person with sudden onset of fever >38°C and cough or sore throat, at the seven sentinel sites involved in influenza surveillance in Yaounde. All the participants were Cameroonian with no history of recent travelling and had never received an antiviral drug against influenza virus. Since this study was a public health program implemented by the Cameroon Ministry of Public Health, there was no need of ethical approval. The samples in 2 ml cryovials containing virus transport medium were kept at 4°C and transported twice per week to the Pasteur Centre of Cameroon (PCC) for analysis. Samples were analyzed by two different techniques: detection of influenza viruses by using a one-step real-time reverse transcription-polymerase chain reaction (RT-PCR) in an ABI Prism 7300 thermocycler (Applied Biosystems) [12] and virus isolation.

### Virus characterization

Viruses were isolated and passaged in Madin-Darby canine kidney (MDCK) cells. Serotyping of virus isolates was performed by hemagglutination inhibition (HI) assay using human group O red blood cells and 2007 reference reagents for influenza virus diagnosis from a WHO kit provided by the Center for Disease Control (CDC), Atlanta. Virus isolates were sent to the WHO Collaborating Center (CC) for Reference and Research on influenza, London, UK for more detailed antigenic characterization, by HI using post-infection ferret antisera, genetic characterization and analysis of antiviral drug resistance.

### Assay of neuraminidase inhibitor susceptibility

Neuraminidase activity was measured using the fluorescent substrate, 2'- (4-methylumbelliferyl)-α-D-N-acetylneuraminic acid (MUNANA; Sigma) [13]. Briefly, 15 μl of virus was incubated with 30 μl of 100 μM MUNANA in 32.5 mM MES buffer pH 6.5 containing 4 mM CaCl_2 _for 1 hr at 37°C. The reaction was stopped by addition of 150 μl 0.14 M NaOH in 83% Ethanol and fluorescence of the released 4-methylumbelliferone was measured at excitation and emission wavelengths of 365 nm and 450 nm, respectively. The activity of each virus sample was titrated, by assaying serial twofold dilutions, and virus suspensions were adjusted to equivalent NA activities, which fell in the linear portion of the activity curve. Each virus was preincubated for 30 minutes at 37°C with oseltamivir or zanamivir at final concentrations of 5 μM-0.05pM, in serial 10-fold dilutions, NA activity measured and the drug concentration that inhibited 50% of the neuraminidase activity (IC_50_) was determined [14].

### Sequence analysis

The primers used for determination of HA, NA and M gene sequences can be obtained on request. Phylogenetic analysis of the nucleotide sequences used maximum parsimony (Phylogenetic Analysis Using Parsimony [PAUP] version 4.0; D Swofford, Illinois Natural History Survey, Champaign, IL, USA).

## Results

Between November 2007 and October 2008, a total of 238 patients with ILI were included in the study. Their mean age was 20.9 years (range 2 months - 86 years). One third of the patients were under 5 and 5% were more than 54 years old and the sex ratio was 1.5 female: male. No patients had been previously vaccinated or treated with anti-influenza drugs.

**Figure 1:**
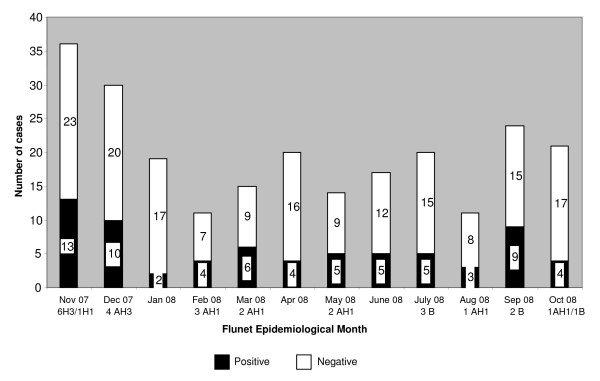
**Numbers of influenza viruses detected among ILI cases each month, by RT-PCR (shaded histogram) and viral isolation (numbers below)**.

Seventy (29%) samples were positive for influenza by RT-PCR, of which only 26 (11%) were positive by virus isolation. By HI assay, 20 of 26 isolates were influenza type A (10 A(H3N2) and 10 A(H1N1)) and 6 were influenza type B (2 B/Victoria/2/87 lineage and 4 B/Yagamata/16/88 lineage). All 10 influenza A(H3N2) strains were isolated during November and December 2007. Only A(H1N1) viruses were isolated from February to June 2008 and all 6 influenza B viruses were isolated between July and October 2008 (Figure 1).

The proportion of influenza virus positive samples per month ranged from 11% to 40%. Although virus was not isolated from samples obtained in January, April or June 2008, virus was detected using the RT-PCR technique throughout the year, in all the isolate-positive samples and some isolate-negative samples (Figure 1).

**Figure 2:**
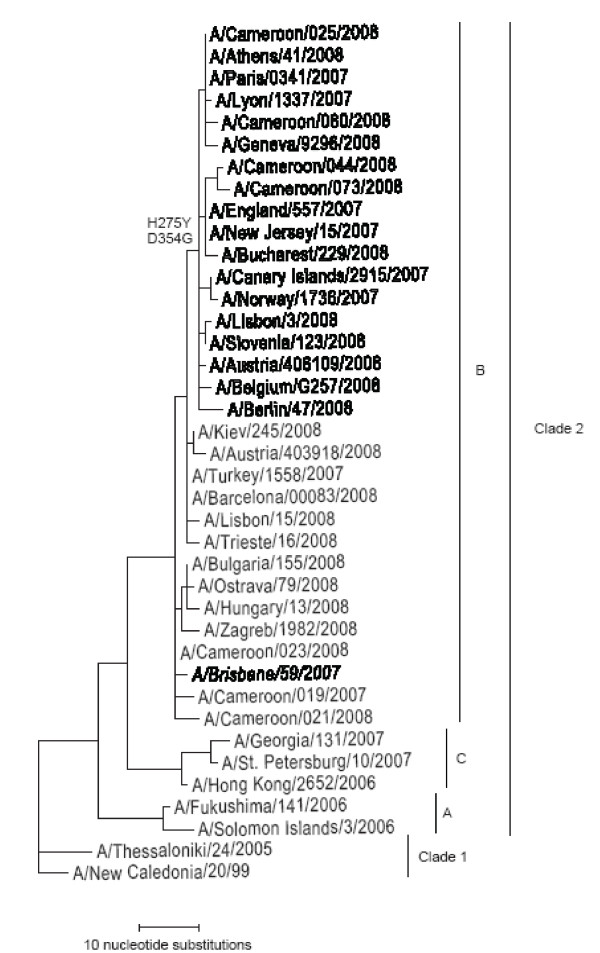
**Phylogenetic comparison of nucleotide sequences of the neuraminidase genes of recent influenza A(H1N1) viruses**. Oseltamivir-resistant viruses are indicated by bold typeface. The vaccine virus A/Brisbane/59/2007 is in bold italics.

The influenza A(H1N1) viruses were subjected to more detailed characterization. In HI tests using post-infection ferret antisera, the ten viruses were shown to be antigenically closely related the prototype vaccine virus A/Brisbane/59/2007. HA sequences of the viruses isolated from November to December confirmed that they were similar to those of other contemporary A/Brisbane/59/2007-like viruses. Analysis of susceptibility to the NA inhibitors oseltamivir and zanamivir showed that the NAs of seven (70%) of the ten A(H1N1) isolates were highly resistant to oseltamivir, with an average IC_50 _of 409 ± 53 nM compared to 1.2 ± 0.3 nM for the three oseltamivir-sensitive viruses. All viruses retained sensitivity to zanamivir. Whereas the three earliest isolates were sensitive, all the H1N1 viruses isolated from the latter half of February onwards were resistant to oseltamivir. Sequences of the NAs of seven of the viruses isolated between November 2007 and May 2008 showed that, whereas those of the sensitive viruses were close to the sequence of A/Brisbane/59/2007, the NAs of the oseltamivir-resistant viruses possessed the substitution of histidine by tyrosine at residue 275 (H275Y), well known to confer a high level resistance to oseltamivir (Figure 2). In addition the resistant NAs were characterised by the substitution of aspartic acid 354 by glycine, typical of the majority of oseltamivir-resistant H1N1 viruses that emerged in Europe and elsewhere during late 2007 and 2008 [15].

## Discussion

This is the first study providing data on human influenza viruses circulating in a Central African country. We observed circulation of both influenza A subtypes, H3N2 and H1N1, and influenza B viruses in the human population in Cameroon during this first year. Some African countries, including Kenya, Mauritius, Senegal, South Africa, Tunisia and Uganda have also reported co-circulation of both types of influenza virus during 2008 [3,4]. By using molecular techniques, influenza virus was detected throughout the year while virus isolation alone failed to detect virus during three months. Molecular techniques are thus suitable for the determination of influenza prevalence. However, since isolation of viruses allows important detailed analyses of their antigenic, genetic and drug susceptibility characteristics, we recommend a combination of both techniques for influenza surveillance.

All 10 influenza A(H3N2) viruses were isolated in Cameroon during November and December 2007 when A(H1N1) viruses were predominant in most northern hemisphere countries, as well as in some African countries [3]. However, during the same period, sporadic activity of influenza A(H3N2) was reported in a number of African countries, including Ghana, Kenya, South Africa, Tunisia and Uganda. The pattern of A(H1N1) virus isolation in Cameroon (February to June) was similar to that in other countries [4] and their antigenic and genetic characteristics were similar to those of contemporary A/Brisbane/57/2007-like viruses isolated in other parts of the world.

Of particular note was the emergence of high frequency resistance to oseltamivir among Cameroonian A(H1N1) isolates in early 2008. Seventy percent of H1N1 isolates were shown to be highly resistant to oseltamivir http://www.who.int/csr/disease/influenza/H1N1200801013.pdf. The resistant viruses isolated in February and March appear to be the first oseltamivir-resistant viruses detected in Central Africa, in relation to those isolated more recently in South Africa [16]. The NA sequences of those viruses, with the D354G substitution in addition to the H275Y mutation, were closely related to the majority of oseltamivir-resistant viruses isolated in other parts of the world during 2008. Furthermore, as observed for the majority of H1N1 viruses isolated elsewhere during late 2008, H1N1 viruses isolated in Cameroon during November and December 2008 were resistant to oseltamivir.

Oseltamivir has never been used to any large extent in Cameroon and there is no evidence that any of the Cameroonian patients were exposed to the drug before or during influenza infection. Therefore, oseltamivir resistance is unlikely to be related to antiviral medication of patients in Cameroon, as in other countries, where there are few instances of resistant viruses being isolated from persons who have either been treated or been in close contact with another individual who has been treated with oseltamivir. It is evident that the oseltamivir-resistant H1N1 viruses can readily transmit between individuals and that they have a selective epidemiological advantage, in the absence of drug pressure, which has promoted their emergence and gradual replacement of the antigeniclly similar oseltamivir-sensitive viruses. Determining the origins and genesis of these drug-resistant viruses, in the absence of drug pressure, will be important in understanding the emergence and persistence of oseltamivir resistance in relation to the evolution of influenza viruses, drug use and pandemic preparedness.

## Conclusions

This study confirmed the circulation of human influenza A(H1N1), A(H3N2) and B viruses in Central Africa and documented the emergence of oseltamivir-resistant H1N1 viruses in Cameroon. Moreover, influenza surveillance is especially important in a region where outbreaks of highly pathogenic A(H5N1) influenza have occurred in domestic poultry. The study also emphasizes the importance of monitoring drug susceptibility as well as the antigenic and genetic characteristics of the influenza viruses as a component of pandemic preparedness.

## Competing interests

The authors declare that they have no competing interests.

## Authors' contributions

SASM and PCa carried out the viral isolation, serological and molecular studies. VG and PCo participated in antigenic and genetic characterization and analyses of antiviral resistance. RN, DNN, AH and DR took part in the design of the study, analysis of the results and writing of the manuscript. All authors read and approved the final version of the manuscript.

## Pre-publication history

The pre-publication history for this paper can be accessed here:

http://www.biomedcentral.com/1471-2334/10/56/prepub

## References

[B1] Glezen WP Emerging infections: pandemic influenza Epidemiol Rev 1996 18 1 64 76 8877331 10.1093/oxfordjournals.epirev.a017917

[B2] Hilleman MR Realities and enigmas of human viral influenza: pathogenesis, epidemiology and control Vaccine 2002 20 25-26 3068 3087 10.1016/S0264-410X(02)00254-2 12163258

[B3] WHO Recommended composition of influenza virus vaccines for use in the 2008-2009 influenza season Wkly Epidemiol Rec 2008 83 9 81 87 18309579

[B4] WHO Recommended composition of influenza virus vaccines for use in the 2009 southern hemisphere influenza season Wkly Epidemiol Rec 2008 83 41 366 372 18846716

[B5] Claas EC Osterhaus AD van Beek R De Jong JC Rimmelzwaan GF Senne DA Krauss S Shortridge KF Webster RG Human influenza A H5N1 virus related to a highly pathogenic avian influenza virus Lancet 1998 351 9101 472 477 10.1016/S0140-6736(97)11212-0 9482438

[B6] Stohr K The global agenda on influenza surveillance and control Vaccine 2003 21 16 1744 1748 10.1016/S0264-410X(03)00065-3 12686087

[B7] Akoua-Koffi C Kouakou B Kadjo H Elia G Koffi SP Adjogoua E Dosso M Ehouman A [Results of two-year surveillance of flu in Abidjan, Cote d'Ivoire] Med Trop (Mars) 2007 67 3 259 262 17784678

[B8] Besselaar TG Botha L McAnerney JM Schoub BD Antigenic and molecular analysis of influenza A (H3N2) virus strains isolated from a localised influenza outbreak in South Africa in 2003 J Med Virol 2004 73 1 71 78 10.1002/jmv.20063 15042651

[B9] Dosseh A Ndiaye K Spiegel A Sagna M Mathiot C Epidemiological and virological influenza survey in Dakar, Senegal: 1996-1998 Am J Trop Med Hyg 2000 62 5 639 643 11289677 10.4269/ajtmh.2000.62.639

[B10] Gachara G Ngeranwa J Magana JM Simwa JM Wango PW Lifumo SM Ochieng WO Influenza virus strains in Nairobi, Kenya J Clin Virol 2006 35 1 117 118 10.1016/j.jcv.2005.10.004 16309952

[B11] Rabarijaona LP Rakotondrarija NT Rousset D Soares JL Mauclere P [Influenza epidemiologic and virologic surveillance in Antananarivo from 1995 to 2002] Arch Inst Pasteur Madagascar 2003 69 1-2 20 26 15678811

[B12] Spackman E Senne D Myers TJ Bulaga L Garber L Perdue M Lohman K Daum L Suarez D Development of a Real-Time Reverse Transcriptase PCR Assay for Type A Influenza Virus and the Avian H5 and H7 Hemagglutinin Subtypes J Clin Microbiol 2002 40 3256 3260 10.1128/JCM.40.9.3256-3260.2002 12202562 PMC130722

[B13] Potier M Mameli L Belisle M Dallaire L Melancon SB Fluorometric assay of neuraminidase with a sodium (4-methylumbelliferyl-alpha-D-*N*-acetylneuraminate) substrate Anal Biochem 1979 94 287 296 10.1016/0003-2697(79)90362-2 464297

[B14] Wetherall N Trivedi T Zeller J Hodges-Savola C McKimm-Breschkin J Zambon M Hayden F Evaluation of Neuraminidase Enzyme Assays Using Different Substrates To Measure Susceptibility of Influenza Virus Clinical Isolates to Neuraminidase Inhibitors: Report of the Neuraminidase Inhibitor Susceptibility Network J Clin Microbiol 2003 41 742 750 10.1128/JCM.41.2.742-750.2003 12574276 PMC149673

[B15] Meijer A Lackenby A Hungnes O Lina B Werf S van der Schweiger B Opp M Paget J Kassteele J van de Hay A Zambon M Oseltamivir-resistant influenza virus A(H1N1), Europe, 2007-08 season Emerg Infect Dis 2009 15 4 552 560 10.3201/eid1504.081280 19331731 PMC2671453

[B16] Besselaar TG Naidoo D Buys A Gregory V McAnerney JM Manamela JM Blumberg L Schoub BD Widespread oseltamivir resistance in influenza A viruses (H1N1), South Africa Emerg Infect Dis 2008 14 11 1809 1810 10.3201/eid1411.080958 18976580 PMC2630761

